# Effects of contralateral nephrectomy timing and ischemic conditions on kidney fibrosis after unilateral kidney ischemia-reperfusion injury

**DOI:** 10.1080/0886022X.2022.2126790

**Published:** 2022-09-26

**Authors:** Junhua Zhang, Ruihua Shen, Hui Lin, Juan Pan, Xinyuan Feng, Ling Lin, Dan Niu, Yanjuan Hou, Xiaole Su, Chen Wang, Lihua Wang, Xi Qiao

**Affiliations:** a Department of Nephrology, Second Hospital of Shanxi Medical University, Taiyuan, People’s Republic of China; b Shanxi Kidney Disease Institute, Taiyuan, People’s Republic of China; c Institute of Nephrology, Shanxi Medical University, Taiyuan, People’s Republic of China; d Department of Pathology, Second Hospital of Shanxi Medical University, Taiyuan, People’s Republic of China

**Keywords:** Acute kidney injury, chronic kidney disease, transition, ischemia-reperfusion injury, the time interval between contralateral nephrectomy and uIRI, ischemic duration, ischemic temperature

## Abstract

Acute kidney injury (AKI) is an important cause of chronic kidney disease (CKD), but the underlying mechanisms are unclear. Animal models are tools for studying the AKI-CKD progression. Kidney ischemia-reperfusion injury (IRI) models, especially the unilateral IRI (uIRI) model with delayed contralateral kidney resection, are commonly used to induce fibrotic progression to CKD after AKI. However, in previous studies, we found that details of the operation had a significant impact on the long-term outcomes of the kidney in this uIRI model. In this study, we investigated the effects of resection timing of the contralateral intact kidney, core body temperatures during ischemia, and time length of kidney ischemia on kidney function, histological injury and kidney fibrosis after AKI, using a mouse uIRI model with delayed contralateral nephrectomy. The results showed that all these parameters significantly affected the AKI-CKD transition. The post-AKI fibrosis worsened and the survival rate declined with a longer interval between contralateral nephrectomy and uIRI, higher ischemic body temperature, or longer ischemic duration when the other two variables were fixed. In conclusion, in the uIRI model with delayed contralateral nephrectomy, kidney fibrosis after AKI is influenced by many factors. Strictly controlling the experimental conditions is very important for the stability and consistency of the model.

## Introduction

The incidence of acute kidney injury (AKI) has increased dramatically worldwide in recent years [1]. AKI can be caused by organ transplantation, postoperative underperfusion, bleeding, dehydration, shock and sepsis in clinical practice [2]. Recent epidemiological and experimental studies have shown that the severity, frequency, and duration of AKI are closely associated with the subsequent incidence of chronic kidney disease (CKD). Kidney fibrosis after AKI can lead to CKD or even end-stage kidney disease (ESKD) [3–5], implying that AKI and CKD are linked syndromes [6]. However, the mechanisms underlying the transition from AKI to CKD remain unknown despite numerous experimental and clinical studies. Animal models are important tools for investigating the mechanisms by which AKI leads to CKD. Kidney ischemia-reperfusion injury (IRI) is one of the major causes of AKI, and several animal models of IRI-induced AKI have been developed [7], including the bilateral IRI (bIRI) model, the unilateral IRI model with intact contralateral kidney, the uIRI model with simultaneous contralateral nephrectomy, and the uIRI model with delayed contralateral nephrectomy.

The bIRI model is less consistent because when the ischemic injury of both kidneys is too mild, there are no significant fibrotic changes in the long term. When AKI is too severe, mice may die in the acute injury phase. Furthermore, small differences in the injury of the two kidneys may lead to significant differences in their chronic kidney pathology after a few weeks [8,9]. The uIRI model with simultaneous contralateral nephrectomy is similar to the bIRI model as only the injured kidney remains due to the other side nephrectomy [10]. In the uIRI model with an intact contralateral kidney, the fibrosis of the ischemic kidney is severe [11,12]. This model is very consistent and reliable for long-term observation by researchers. However, due to the presence of contralateral undamaged kidney, it cannot be used for the evaluation of estimated glomerular filtration rate (eGFR), serum creatinine (SCr), and blood urea nitrogen (BUN) [11–14]. Compared with the two uIRI models mentioned above, the uIRI model with delayed contralateral nephrectomy overcomes these problems and can be used for both long-term observations after AKI and kidney function monitoring. Therefore, this model is suitable for studying AKI-CKD transition [15].

Nevertheless, different ischemic conditions and the interval between contralateral nephrectomy and uIRI may have significant impacts on the progression and severity of CKD after AKI. Previous studies have focused on the effect of the ischemic duration on post-AKI kidney fibrosis and have not paid much attention to the core body temperature during ischemia. For example, Skrypnyk et al. [12] controlled the ischemic temperature at 38 °C by water bath system, with the ischemic duration of 30 min, and the contralateral intact kidney was removed 8 days after uIRI. The result showed that the ischemic kidney had significant fibrotic changes 28 days after uIRI. With the same ischemic duration, Xiao et al. [8] showed that significant fibrotic changes happened 11 days after uIRI when the ischemic temperature was controlled at 37 °C − 38 °C and the time interval between uIRI and contralateral nephrectomy was 10 days. Some researchers, such as Colombaro et al. [14] and Li et al. [16] did not mention the ischemic temperature in their studies.

Meanwhile, few people researched the effect of the time interval between contralateral nephrectomy and uIRI in this model, resulting in widely varying results among different laboratories, which also dramatically affected subsequent experiments. In this study, we investigated the effects of the main factors affecting kidney fibrosis after AKI, including the time interval between contralateral nephrectomy and uIRI, the core body temperature during ischemia, and the ischemic duration. We aimed to provide a reference to make an appropriate animal model for studying the AKI-CKD transition.

## Materials and methods

### Animal models

Male C57BL/6J mice (8–10 weeks of age, approximately 22 g of weight; GemPharmatech Co., Ltd., Nanjing, China) were provided with free access to food and water in the Animal Experiment Center of Shanxi Medical University. The breeding conditions were as follows: humidity 40–70%, temperature 23 °C, air cleanliness class 7, controlled 12-h light/dark cycle under pathogen-free conditions, feed Co60 irradiation, and purified water. All procedures for this study were approved by the Animal Ethics Committee of the Second Hospital of Shanxi Medical University (ethics number: DW2022028).

### Protocols

Considering that ischemic durations, core body temperatures during ischemia, and resection timings of the contralateral uninjured kidney may all have impacts on the severity of AKI and subsequent kidney fibrosis, we investigated the effects of the above three factors individually, with the other two fixed. Three experiments were conducted as follows.

**Figure 1.**
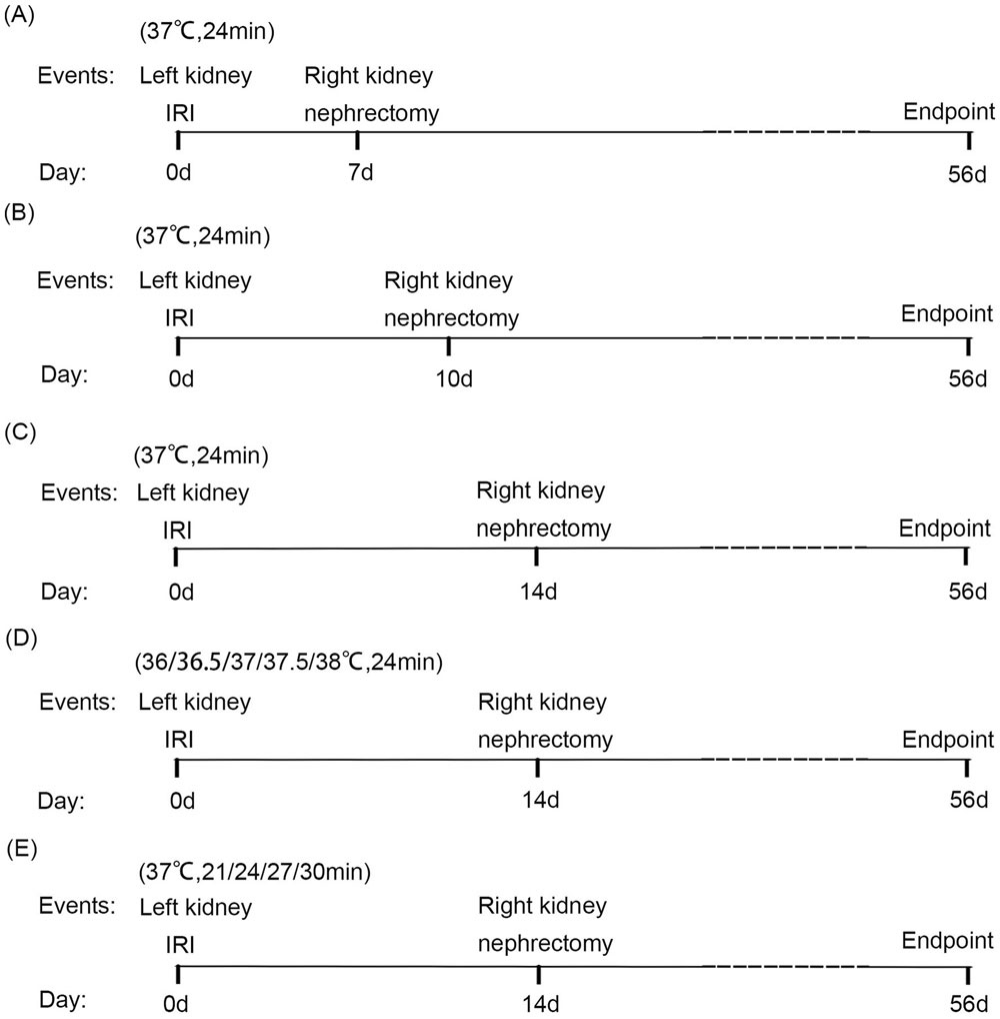
Protocols of the unilateral ischemia-reperfusion injury (uIRI) with delayed contralateral nephrectomy. (A–C): The left kidney was subjected to IRI on day 0, and right kidney resection was conducted at different time points (day 7, day 10, and day14) with the ischemic duration fixed at 24 min with the core body temperature fixed at 37 °C during kidney ischemia. (D): The left kidney was subjected to IRI on day 0, and right kidney resection was conducted under different core body temperatures (36 °C, 36.5 °C, 37 °C, 37.5 °C, and 38 °C) with the ischemic time of the left kidney fixed at 24 min, and the right kidney was removed 14 days after the left kidney IRI. (E): The left kidney was subjected to IRI on day 0, and right kidney resection was conducted at different ischemic durations (21 min, 24 min, 27 min, and 30 min) with the core body temperature fixed at 37 °C during kidney ischemia, and the right kidney was removed 14 days after the left kidney IRI. Kidney weight, kidney function, kidney histological injury, and kidney fibrosis were assessed until day 56.

Protocol 1: To observe the effect of the interval between contralateral nephrectomy and uIRI on the kidney function and post-AKI fibrosis, the ischemic time of the left kidney was fixed at 24 min and the core body temperature was fixed at 37 °C during the kidney ischemia meanwhile. The right kidney resection was conducted on day 7, day 10, and day 14, respectively (*n* = 8 per group) after the left kidney IRI. We determined the duration of ischemia and kidney resection based on the results of previous studies [8,10,12,15] and our pre-experimental results. See Figure 1(A–C) for the study protocol.

Protocol 2: To observe the effect of core body temperature during ischemia on the AKI-CKD transition, the ischemic time of the left kidney was fixed at 24 min, and the right kidney was removed 14 days after the left kidney IRI. The core body temperature was controlled at 36 °C, 36.5 °C, 37 °C, 37.5 °C, and 38 °C, respectively (*n* = 8 per group). See Figure 1(D) for the study protocol.

Protocol 3: To observe the effect of kidney ischemic durations on the transition from AKI to CKD, the core body temperature was fixed at 37 °C during ischemia, and the right kidney was removed 14 days after the left kidney IRI. We chose this time point was because kidney fibrosis, which was the typical changes after AKI to CKD, was very obvious according to the reports [8,10,12,15] and our pre-experimental results. The ischemic time of the left kidney was controlled at 21 min, 24 min, 27 min and 30 min, respectively (*n* = 8 per group). See Figure 1(E) for the study protocol.

All mice were euthanized at 8 weeks after IRI of the left kidney. Part of the left kidney tissue was stored at −70 °C for the subsequent RT-PCR and western blot analysis, and the other part was fixed in neutral formalin for histopathological analysis. Blood samples were collected *via* the retro-orbital sinus for biochemical testing.

### Surgical procedure

Before surgery, all mice fasted for 12 h with free access to water and were injected with 3% pentobarbital sodium 50 mg/kg intraperitoneally. During anesthesia, the rectal temperature was maintained at 36 °C, 36.5 °C, 37 °C, 37.5 °C, or 38 °C, respectively, in different experimental groups. To achieve this, the mouse was placed with its back on a heating pad (CW-26, BARBAROUS GROWTH, Zhejiang, China) in a position with its head and neck extended to ensure that its airway remained unobstructed, and the eyes were covered with warm saline gauze to avoid drying during the procedure. After anesthesia, the hair on the left back of the mouse was shaved with the hair clipper. The skin in the surgical area was then wiped clean with a 75% alcohol swab. Generally, the body temperature of the mouse drops after hair shaving. The surgical procedure was carried out when the core body temperature became constant, which could take about 15 min. The left kidney could be seen by cutting the left dorsal skin 0.5 cm next to the midline and 0.5 cm at the lower edge of the rib cage of the mouse. Then we squeezed the left abdomen with one hand to make the left kidney out of the body cavity, lifted the kidney with blunt curved forceps (taking care to avoid damaging the kidney), detached the kidney pedicle and clamped it quickly, and placed the clamped kidney under the skin to keep it warm and moisturized. Successful ischemia was characterized by a gradual color change of the kidney from red to dark purple, which lasted 21 min, 24 min, 27 min and 30 min respectively, in different experimental groups. The kidney color changed back to red within 2–5 min after releasing the clamp. Then we closed the abdominal cavity with layered sutures. The right dorsal skin and muscle were incised on day 7, day 10, and day 14 after uIRI, respectively, in different experimental groups, and the right kidney was resected. Then close the wound with standard sutures. After each surgery, 500 μL saline was injected intraperitoneally to compensate for the fluid loss during surgery. At the same time, the mouse was kept on a heating pad for about 2–3 h until it gained full consciousness before being transferred to the housing cage. The sham group did not clamp the kidney pedicle, and the rest of the surgical operations were the same as those of the uIRI group. All surgical operations were performed by the same person with more than 2 years of experience in the operation of this model.

### Kidney function assay

SCr levels were measured by the Creatinine Kit (Jaffe Method, 100020170, Biosino Biotechnology and Science). BUN levels were measured by the Urea Kit (Urease-Glutamate Dehydrogenase Method, Biosino Biotechnology and Science).

### Kidney weight assay

After urination, the body weights of mice were measured by a precision electronic scale. Fresh kidneys were rinsed three times in pre-chilled saline, then excess water on the kidneys was blotted off with filter paper. The kidney capsules were peeled off on the ice and the kidneys were weighed immediately. The ratio of kidney weight to body weight was calculated to evaluate corrected kidney weight in mg/g.

### Hematoxylin-eosin (HE) staining and kidney injury score

Neutral formalin-fixed kidney tissues were routinely dehydrated, embedded in paraffin, sectioned (3 μm) and stained with HE to observe the damage of kidney tissues. Ten randomly selected fields of the kidney cortex of each section were captured with light microscopy at 400× magnification. Two pathologists scored the injury to the glomerulus and kidney tubules separately. The tubular injury was scored on a scale of 0–4 using the following criteria [17]: 0, no injury; 1, affecting 1–25% of the kidney area; 2, affecting 26–50% of the kidney area; 3, affecting 51–75% of the kidney area; and 4, affecting 75–100% of the kidney area. The degree of tubular injury was evaluated semi-quantitatively by calculating the area of kidney tubular injury. The glomerular injury was evaluated and scored on a scale of 0–4 according to the percentage area of mesangial expansion or sclerosis using the following criteria [17]: 0, normal glomeruli; 1, 1–25% of area injured; 2, 26–50% of area injured; 3, 51–75% of area injured; and 4, 76–100% of area injured. The final glomerular damage score = 0 × (% grade 0 glomeruli) + 1 × (% grade 1 glomeruli) + 2 × (% grade 2 glomeruli) + 3 × (% grade 3 glomeruli) + 4 × (% grade 4 glomeruli). More than 50 glomeruli were scored per mouse.

### Masson’s trichrome staining

Neutral formalin-fixed kidney tissues were routinely dehydrated, embedded in paraffin, sectioned (3 μm) and stained with Masson’s trichrome to observe kidney fibrosis. In Masson’s trichrome-stained slices, twenty non-overlapping fields of kidney cortex were captured under 200× magnification. Image-Pro Plus Image Analysis Software was used for automatic measurement and analysis. The percentage of the fibrotic area was used to figure out the level of kidney interstitial fibrosis [8].

### RT-PCR

Total mRNA was extracted from the kidney tissue of each group by Trizol and its concentration was measured, then it was converted to cDNA (Taka Reverse Transcription Kit). The Taqman fluorescence method (TB Green Premix Ex Taq II Real-time RT-PCR) was used to quantify fibronectin (FN) and collagen I (COL-I) RNA expressions in kidney tissues. Specific primers were used for FN (forward: 5′-GGAGTGGCACTGTCAACCTC-3′ and reverse: 5′-ACTGGATGGGGTGGGAAT-3′), and COL-I (forward: 5′-ACATGTTCAGCTTTGTGGACC-3′ and reverse: 5′-TAGGCCATTGTGTATGCAGC-3′) [18] (provided by Sangon Biotech Co., Ltd., Shanghai, China). The amplification conditions are as follows: 95 °C for 30 s, 95 °C for 5 s, 60 °C for 30 s, 40 cycles; 95 °C for 10 s, 65 °C for 5 s, and 95 °C/5 °C. After analyzing the amplification curves and solubility curves of the products, the relative mRNA levels were determined by the comparative CT method (2-ΔΔCt) with 18SrRNA (forward: 5′- CGGACACGGACAGGATTGACAG-3′ and reverse: 5′-AATCGCTCCACCAACTAAGAACGG-3′) as the internal reference.

### Western blot

Radio-immuno-precitation-assay (RIPA) lysis buffer was used to lyse the kidney tissues and the concentration of protein was determined by the BCA method. Gradient gel electrophoresis was used to separate the proteins from 50 μg of samples. Then the proteins were transferred to a nitrocellulose membrane. After blocking with 5% nonfat milk for 2 h, the membranes were blotted overnight at 4 °C with rabbit anti-mouse monoclonal antibody against α-smooth muscle actin (α-SMA) (1:1000, No.19245, Cell Signaling Technology), FN (1:1000, No.ab2413, ABCam), or COL-I (1:1000, No.72026, Cell Signaling Technology), respectively. On the next day, the membranes were rewarmed at room temperature for 0.5 h. After being washed with TBST, the membranes were incubated with HRP Conjugated AffiniPure Goat Anti-rabbit IgG(H + L)(1:1000, No.BA1054, BOSTER Biological Technology) for 2 h. After being washed with TBST again, they were stained with ultra-high-sensitivity ECL chemiluminescence reagent to visualize the immunoblots. β-actin was measured on the same membrane as the internal reference. Protein expressions were determined by quantifying the relative expressions of target proteins versus β-actin.

### Data and statistical analysis

All data examined are expressed as mean ± standard deviation. Comparisons between two groups were accessed by an independent *t*-test. Comparisons between multiple groups were accessed by one-way ANOVA when the data distribution is normal, and the variance is homogeneous, Welch ANOVA was used to assess statistical significance when the variance was heterogeneous, followed by Tukey’s *post hoc* test for multiple comparisons. All statistical analyses were conducted by the GraphPad Prism 8.0 software. *p* < 0.05 was considered to be statistically significant.

## Results

### Protocol 1: effects of the interval between contralateral nephrectomy and uIRI on AKI-CKD transition

#### Survival rate declined with the extension of the interval between contralateral nephrectomy and uIRI

**Figure 2.**
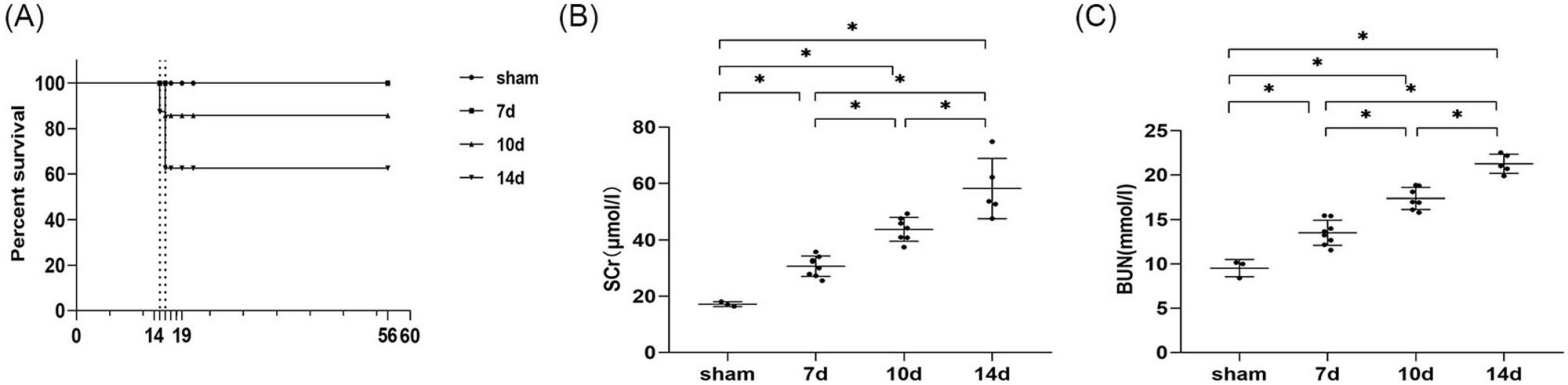
Effects of time intervals between contralateral nephrectomy and unilateral ischemia-reperfusion injury (uIRI) on survival rate and kidney function. The left kidney was subjected to IRI on day 0, and right kidney resection was conducted at different time points (day 7, day 10, and day 14) with the ischemic duration fixed at 24 min with the core body temperature fixed at 37 °C during kidney ischemia. (A): The survival of the mice in each group was scored and presented as a percentage. (B and C): Kidney function was estimated by serum creatinine (SCr), and blood urea nitrogen (BUN). *n* = 5–8/group. *p < 0.05.

As mentioned in experiment 1, the ischemic duration was fixed at 24 min with a core body temperature of 37 °C in uIRI. We observe the effect of contralateral nephrectomy timing after uIRI on the survival of mice. Data showed that in the day 7 group (contralateral intact kidney was resected 7 days after uIRI), all eight mice survived. For the day 10 group, one mouse died the day after contralateral nephrectomy, with a survival rate of 87.5%. For the day 14 group, one mouse died on the first day after the contralateral nephrectomy, and two mice died two days after the contralateral nephrectomy, with a survival rate of 62.5%. These results indicated that the survival rate of mice gradually decreased with the extension of contralateral nephrectomy when the other two influencing factors were fixed (Figure 2(A)).

#### Kidney function declined with the extension of the interval between contralateral nephrectomy and uIRI

Compared with the sham control, SCr and BUN were significantly higher in all mice after contralateral nephrectomy (*p* < 0.05), indicating that they all suffered from kidney damage. Moreover, SCr and BUN levels increased with the extension of the interval between contralateral nephrectomy and uIRI (*p* < 0.05) (Figure 2(B,C)).

#### Kidney histological injury worsened with the extension of the interval between contralateral nephrectomy and uIRI

**Figure 3.**
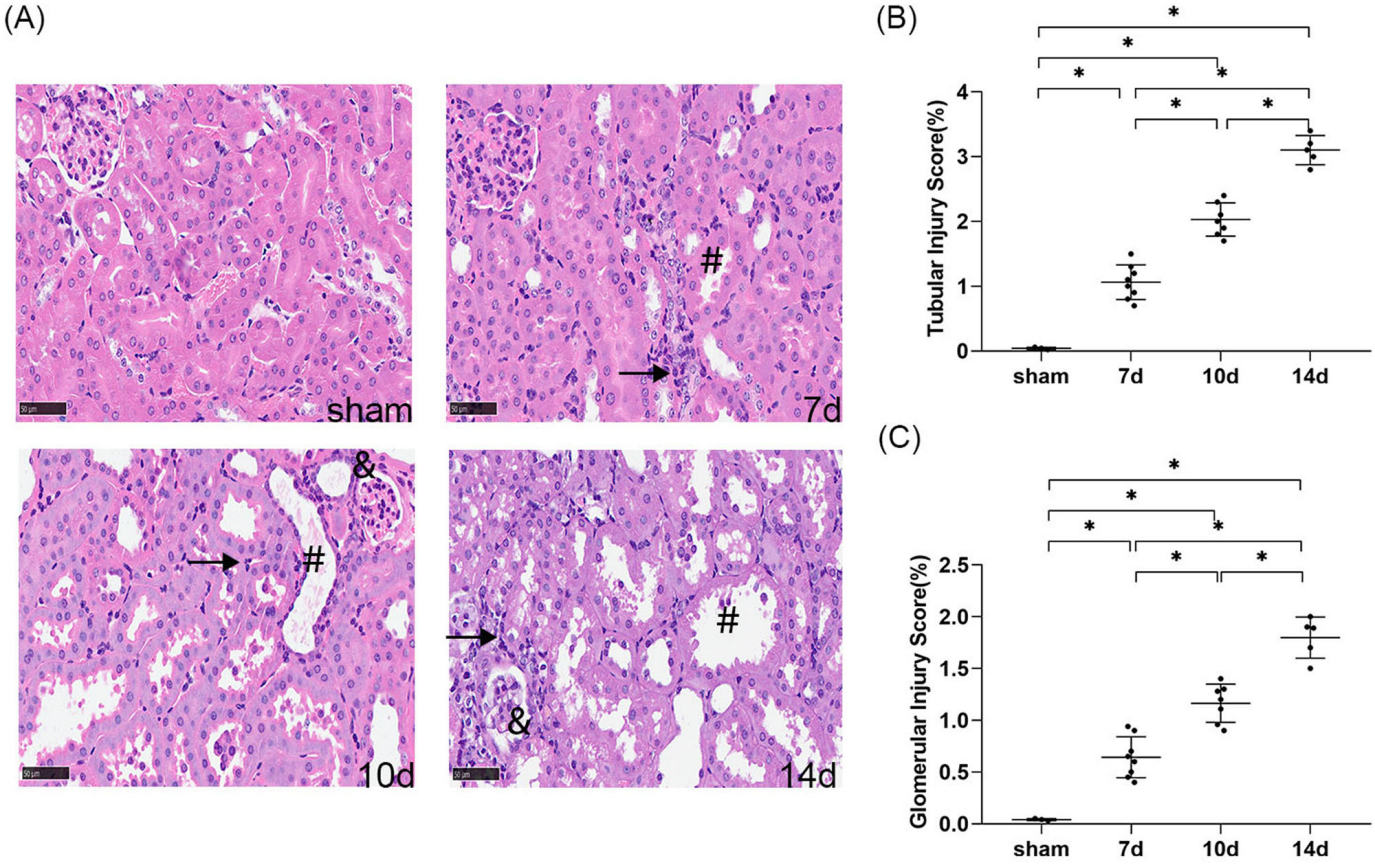
Effects of time intervals between contralateral nephrectomy and unilateral ischemia-reperfusion injury (uIRI) on kidney histological injury. The left kidney was subjected to IRI on day 0, and right kidney resection was conducted at different time points (day 7, day 10, and day14) with the ischemic duration fixed at 24 min with the core body temperature fixed at 37 °C during kidney ischemia. Kidney histological injury was determined by hematoxylin-eosin (HE) staining. (A): Representative HE staining of the injured kidney, which showed the typical histopathological features of post-AKI pathological changes, including different degrees of tubular epithelial cell vacuolar degeneration, brush border detachment, focal disintegration necrosis, dilatation of some tubular lumen (#), kidney tubular epithelial cell edema with variable morphology, and inflammatory cell infiltration (↗). Some glomeruli were wrinkled and sclerotic, with mesangial cell hyperplasia (&) and dilated Bowman’s capsule (magnification, 400×). (B): Semi-quantitation of tubular injury. (C): Semi-quantitation of glomerular injury. *n* = 5–8/group. *p < 0.05.

HE staining demonstrated that the kidney tissue of the sham group was structurally normal at 8 weeks after uIRI. In all uIRI groups, several post-AKI pathological changes were detected in the kidney (Figure 3(A)), including different degrees of tubular epithelial cell vacuolar degeneration, brush border detachment, focal disintegration necrosis, dilatation of some tubular lumen, and kidney tubular epithelial cell edema with variable morphology. Besides, some glomeruli were wrinkled and sclerotic, with mesangial cells hyperplasia and dilated Bowman’s capsule. Inflammatory cell infiltration was also seen in the kidney interstitial. The tubular and glomerular pathological injury scores of the injured kidney were higher compared to the sham (*p* < 0.05), indicating that uIRI with contralateral nephrectomy significantly damaged the kidney. The kidney pathological injury scores increased with the extension of contralateral nephrectomy (*p* < 0.05) (Figure 3(B,C)). These findings indicate that kidney pathological damage gradually increased with the extension of contralateral nephrectomy after uIRI.

#### Kidney fibrosis worsened with the extension of the interval between contralateral nephrectomy and uIRI

##### Kidney weight

**Figure 4.**
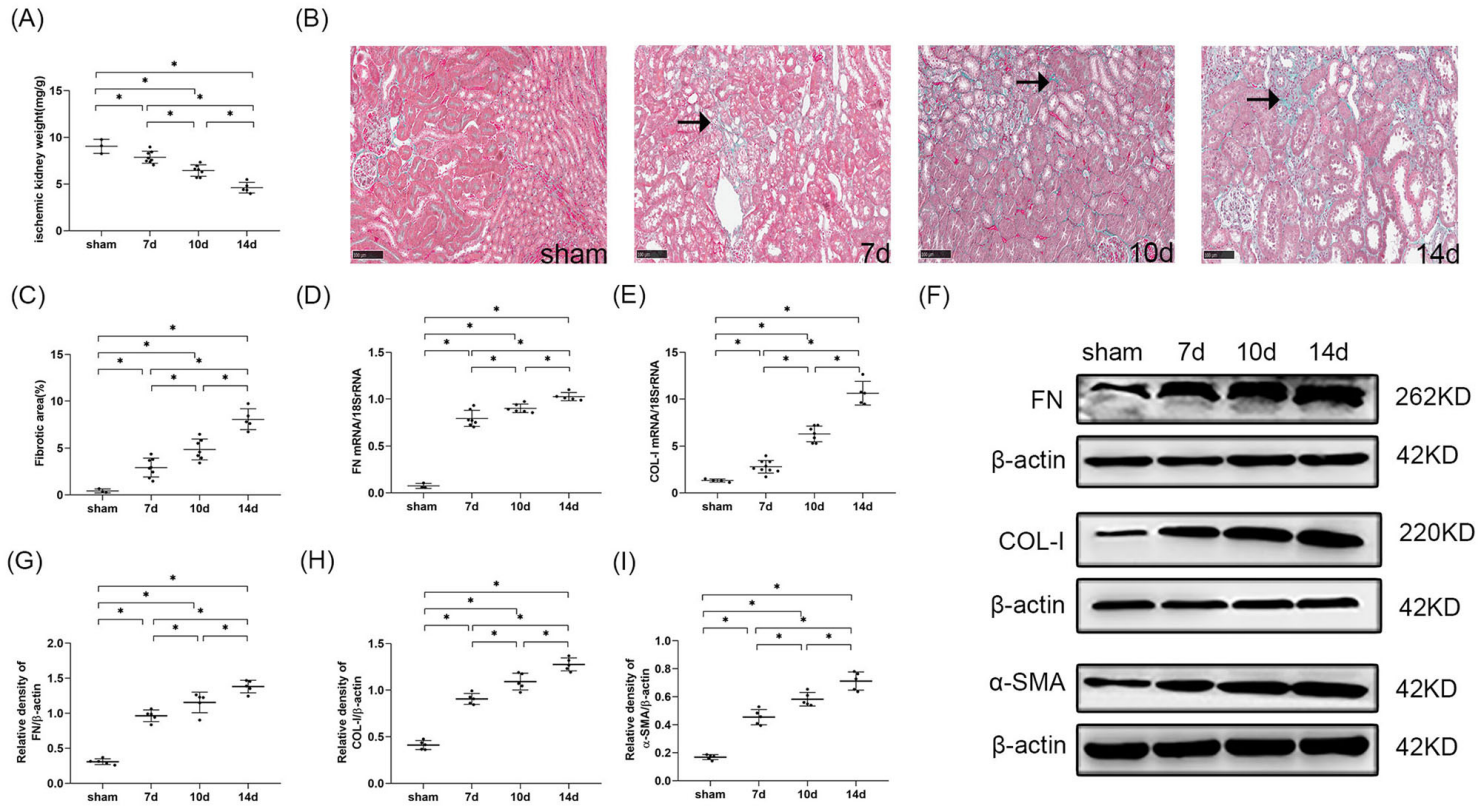
Effects of time intervals between contralateral nephrectomy and unilateral ischemia-reperfusion injury (uIRI) on kidney fibrosis. The left kidney was subjected to IRI on day 0, and right kidney resection was conducted at different time points (day 7, day 10, and day14) with the ischemic duration fixed at 24 min with the core body temperature fixed at 37 °C during kidney ischemia. Kidney fibrosis was assessed by kidney weight, Masson’s trichrome staining, and fibronectin (FN), collagen I (COL-I) and α-smooth muscle actin (α-SMA) expressions. (A): Kidney weight to body weight. (B): Representative Masson’s trichrome staining of the injured kidney, which showed the typical of fibrotic changes (↗) (magnification, 200×). (C): Semi-quantitation of fibrotic area detected by Masson’s trichrome staining. (D): Quantitative RT-PCR analyses for FN expression in the kidney. (E): Quantitative RT-PCR analyses for COL-I expression in the kidney. (F): Representative western blot images for FN, COL-I and α-SMA. (G): Bar chart showing the FN protein expression normalized by β-actin. (H): Bar chart showing the COL-I protein expression normalized by β-actin. I: Bar chart showing the α-SMA protein expression normalized by β-actin. *n* = 5–8/group. *p < 0.05.

The transformation from AKI to CKD is typically characterized by kidney fibrosis [19] and a decrease in kidney weight is a macroscopic indicator of kidney fibrosis [2]. So we measured kidney weight (kidney weight calibrated by body weight) to preliminarily investigate the effect of time intervals between uIRI and delayed contralateral nephrectomy on the AKI-CKD transition. Compared with the sham group, all uIRI groups showed a significant reduction in kidney weight (*p* < 0.05), indicating that all mice in the uIRI groups had kidney fibrosis. The difference in kidney weight changed significantly when the difference in the time interval between contralateral nephrectomy and uIRI was 3 days, and the kidney weight decreased gradually as the interval time increased (Figure 4(A)).

##### Masson’s trichrome stain

Masson’s trichrome staining was performed to further observe the effect of time intervals between contralateral nephrectomy and uIRI on kidney fibrosis. No significant kidney interstitial fibrosis was seen in the sham group, while varying degrees of fibrotic changes in the kidney of all uIRI groups were observed at 8 weeks after uIRI (Figure 4(B)). The semi-quantitative analysis of the fibrotic area showed that the fibrotic area of the kidney in the uIRI groups was significantly increased compared with that in the sham group (*p* < 0.05), indicating that the AKI-CKD transition model was successfully performed. Moreover, with the delay of the contralateral nephrectomy, the kidney fibrosis became increasingly severe (*p* < 0.05), indicating that the kidney fibrosis degree changed significantly when the interval between the contralateral nephrectomy and uIRI was 3 days. With the prolonged time interval between contralateral nephrectomy and uIRI, the kidney fibrosis degree became more aggravated (Figure 4(C)).

##### Extracellular matrix (ECM) accumulation

Kidney interstitial fibrosis is characterized by the deposition of ECM [2], such as FN and COL-I, in the interstitial area of the kidney. Western blot and RT-PCR analysis showed that the expressions of FN and COL-I in the kidney were significantly increased in all uIRI groups compared with the sham group (*p* < 0.05), indicating that all the kidneys underwent fibrotic changes. The expression levels of FN and COL-I in the kidney were higher with the delay of the contralateral nephrectomy (*p* < 0.05) (Figure 4(D–H)).

##### Myofibroblasts in kidney

Kidney fibrosis is characterized by myofibroblast accumulation, and subsequent ECM production [20]. α-SMA was used to detect myofibroblast quantity at 8 weeks after uIRI. Western blot analysis showed that the expression of α-SMA in the kidney tissues significantly increased in all uIRI groups compared with the sham group (*p* < 0.05), indicating that myofibroblasts were pronouncedly more in the kidney of all injured mice. The expression of α-SMA was higher with the delay of contralateral kidney resection (*p* < 0.05) (Figure 4(F,I)). These results showed that the myofibroblasts in the uIRI kidney tissue changed significantly in the 3 days between uIRI and the contralateral nephrectomy, and that the myofibroblasts gradually increased with longer intervals.

### Protocol 2: effects of core body temperatures during ischemia on AKI-CKD transition

#### Survival rate declined with the increase in core body temperature during ischemia

**Figure 5.**
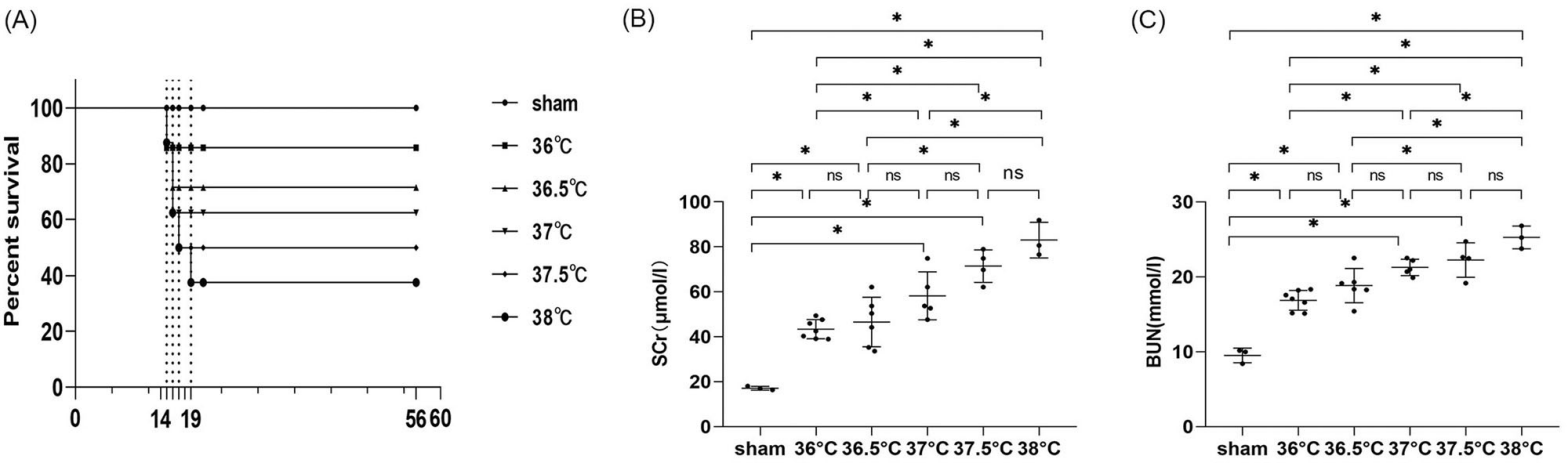
Effects of core body temperatures during ischemia on survival rate and kidney function in mice with uIRI and delayed contralateral nephrectomy. The left kidney was subjected to IRI on day 0, and right kidney resection was conducted under different core body temperatures (36 °C, 36.5 °C, 37 °C, 37.5 °C, and 38 °C) with the ischemic time of the left kidney fixed at 24 min, and the right kidney was removed 14 days after the left kidney IRI. (A): The survival of the mice in each group was scored and presented as a percentage.(B and C): Kidney function was estimated by serum creatinine (SCr), and blood urea nitrogen (BUN). *n* = 3-7/group. *p < 0.05. NS: non-significant.

As mentioned in experiment 2, in order to investigate the effect of core body temperatures during ischemia on AKI-CKD transition, the ischemia duration was fixed at 24 min and the contralateral intact kidney was removed 14 days after uIRI. Data showed that in the 36 °C group (the core body temperature of mice during ischemia was controlled at 36 °C), one mouse died the first day after the contralateral nephrectomy, and the survival rate was 87.5%. For the 36.5 °C group, two mice died in the first 2 days after the contralateral nephrectomy, with 75% of survival rate. Compared with the 36.5 °C group, there was one more mouse died on the second day after contralateral nephrectomy with a survival rate of 62.5% in the 37 °C group. In the 37.5 °C group, except for the three mice that died in the first 2 days after contralateral nephrectomy, one mouse died 3 days after contralateral nephrectomy, with a survival rate of 50%. In addition to what happened in the 37.5 °C group, one mouse died 5 days after the contralateral nephrectomy in the 38 °C group, and the survival rate was 37.5%. These results indicated that the survival rate of mice gradually decreased with the increase in core body temperature during ischemia (Figure 5(A)).

#### Kidney function declined with the increase in core body temperature during ischemia

Compared with the sham group, uIRI resulted in a significant increase in SCr and BUN in all uIRI groups (*p* < 0.05), suggesting that all mice in uIRI groups developed kidney impairment after contralateral nephrectomy. Comparisons between two groups with adjacent temperatures showed that the SCr and BUN of mice increased with an increase of 0.5 °C in ischemic body temperature, but there was no statistical difference (*p* > 0.05), suggesting there were less pronounced changes in the kidney function with a core temperature difference in 0.5 °C during ischemia. However, SCr and BUN significantly increased when core body temperature increased by 1 °C or above during ischemia (*p* < 0.05), suggesting that the kidney function significantly changed with a 1 °C difference in core body temperature during ischemia. Kidney function declined more significantly with increasing ischemic body temperature (Figure 5(B,C)).

#### Kidney tissue injury worsened with increased core body temperature during ischemia

**Figure 6.**
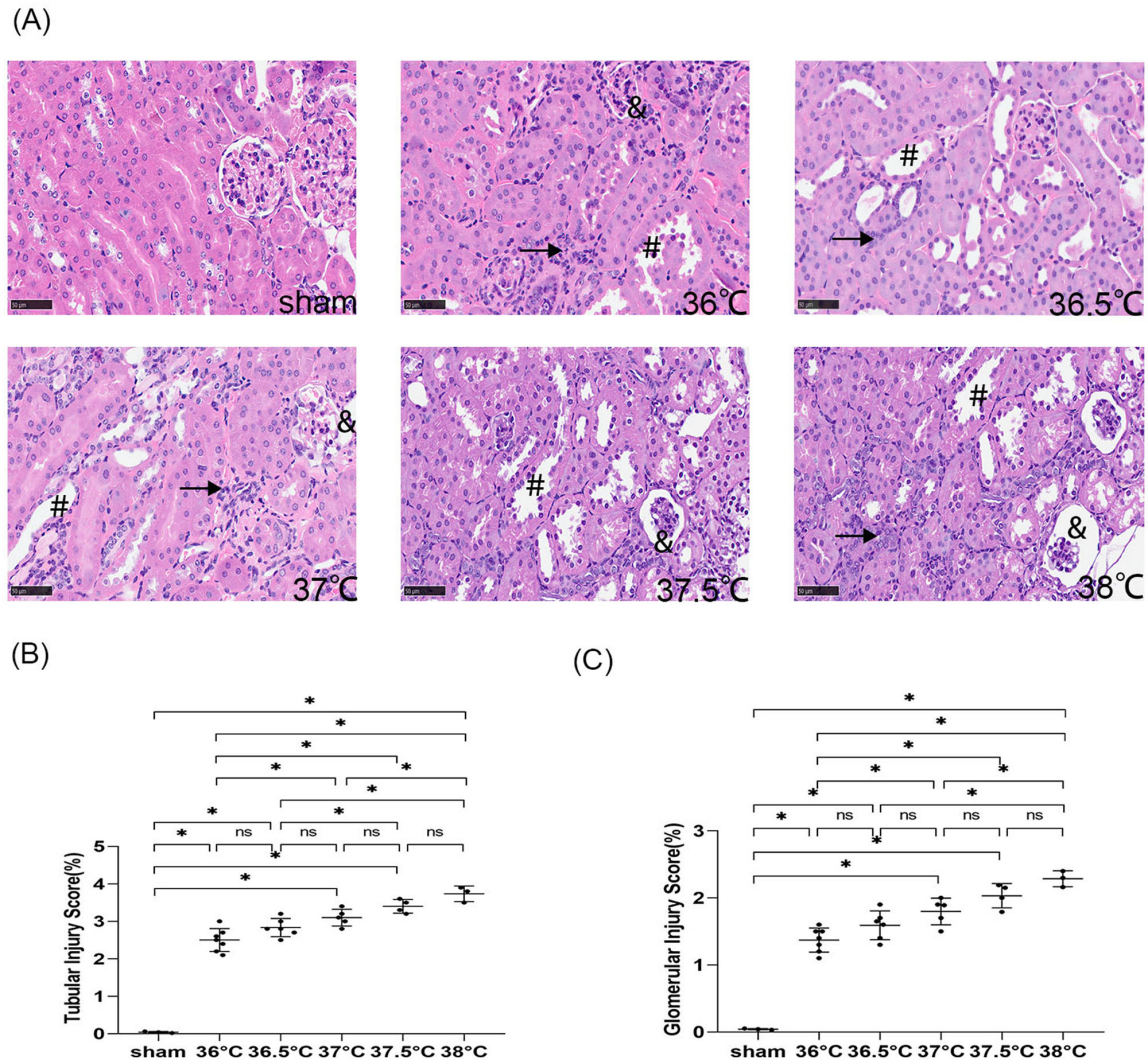
Effects of core body temperatures during ischemia on kidney histological injury in mice with uIRI and delayed contralateral nephrectomy. The left kidney was subjected to IRI on day 0, and right kidney resection was conducted under different core body temperatures (36 °C, 36.5 °C, 37 °C, 37.5 °C, and 38 °C) with the ischemic time of the left kidney fixed at 24 min, and the right kidney was removed 14 days after the left kidney IRI. Kidney histological injury was determined by hematoxylin-eosin (HE) staining. (A): Representative HE staining of the injured kidney, which showed the typical histopathological features of post-AKI pathological changes, including different degrees of tubular epithelial cell vacuolar degeneration, brush border detachment, focal disintegration necrosis, dilatation of some tubular lumen (#), kidney tubular epithelial cell edema with variable morphology, and inflammatory cell infiltration (↗). Some glomeruli were wrinkled and sclerotic, with mesangial cell hyperplasia (&) and dilated Bowman’s capsule (magnification, 400×). (B): Semi-quantitation of tubulointerstitial injury. (C): Semi-quantitation of glomerular injury. *n* = 3–7/group. *p < 0.05. NS: non-significant.

According to HE staining, the kidney tissue structure of the mice in the sham group was normal. IRI-induced pathological changes were observed in all uIRI groups, such as different degrees of tubular epithelial cell vacuolar degeneration, brush border detachment, focal disintegration necrosis, dilatation of some tubular lumen, and kidney tubular epithelial cell edema with variable morphology, as well as wrinkled and sclerotic glomeruli, mesangial cells hyperplasia and dilated Bowman’s capsule, inflammatory cell infiltration in kidney interstitial (Figure 6(A)).

The pathological injury scores of kidney tubules and glomeruli showed that compared with the sham group, the pathological injury of the kidney in all uIRI groups was significantly more severe (*p* < 0.05), suggesting that obvious pathological injury of the kidney appeared in all uIRI groups and that the AKI-CKD model was successfully developed. Comparison between two groups with adjacent temperatures showed that there was an increase in kidney pathological injury scores with a 0.5 °C increase in core body temperature during ischemia, but it had no statistical significance (*p* > 0.05), while kidney pathological injury significantly increased with an increase in core body temperature of 1 °C and above during ischemia (*p* < 0.05) (Figure 6(B,C)). These results indicated that the histopathological damage of the kidney changed significantly with a 1 °C difference in core body temperature during ischemia and gradually increased with the increased core body temperature during ischemia.

#### Kidney fibrosis worsened with the increase in core body temperature during ischemia

##### Kidney weight

**Figure 7.**
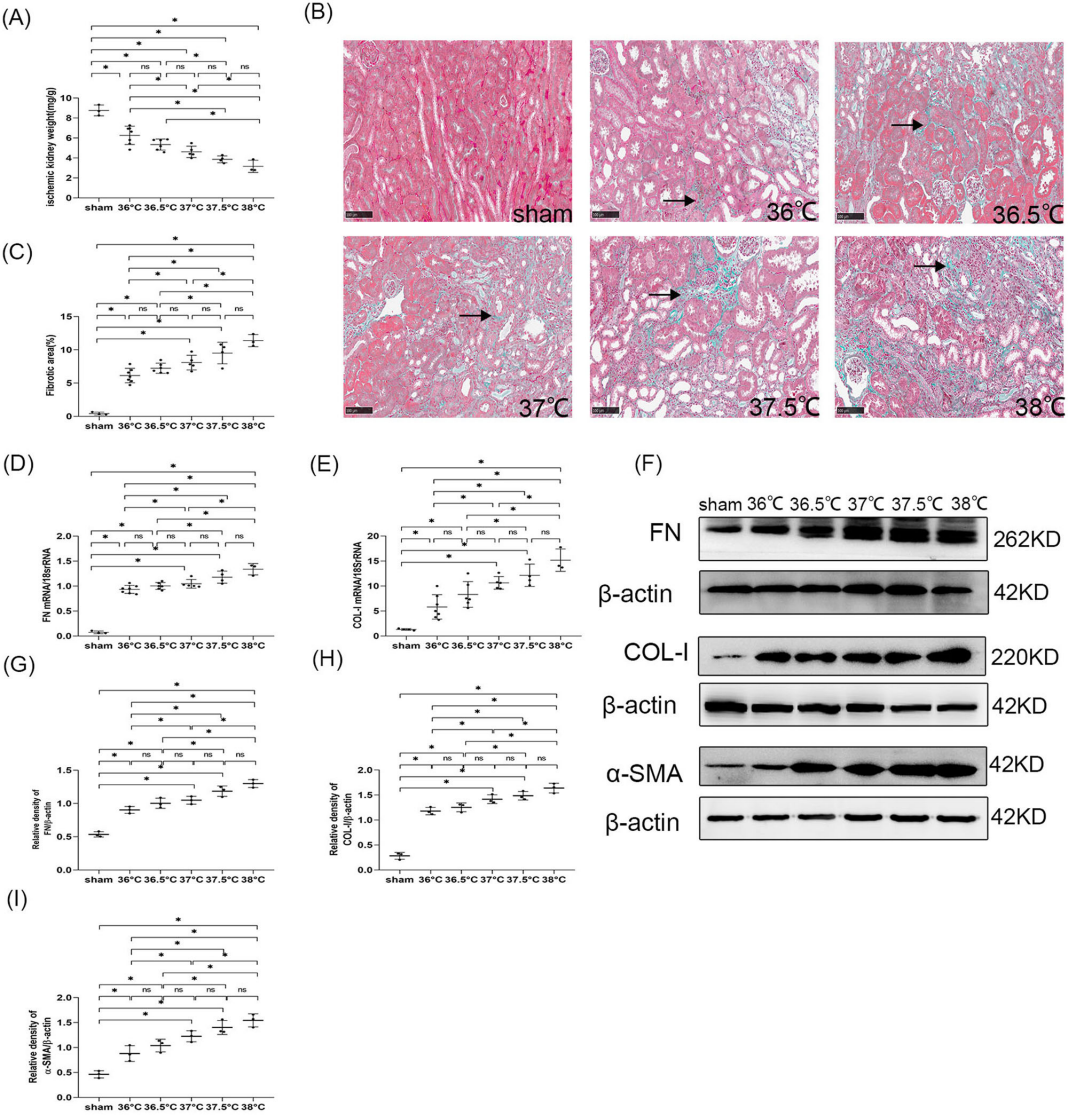
Effects of core body temperatures during ischemia on kidney fibrosis in mice with uIRI and delayed contralateral nephrectomy. The left kidney was subjected to IRI on day 0, and right kidney resection was conducted under different core body temperatures (36 °C, 36.5 °C, 37 °C, 37.5 °C, and 38 °C) with the ischemic time of the left kidney fixed at 24 min, and the right kidney was removed 14 days after the left kidney IRI. Kidney fibrosis was assessed by kidney weight, Masson’s trichrome staining, and fibronectin (FN), collagen I (COL-I) and α-smooth muscle actin (α-SMA) expressions. (A): Kidney weight to body weight. (B): Representative Masson’s trichrome staining of the injured kidney, which showed the typical of fibrotic changes (↗) (magnification, 200×). (C): Semi-quantitation of fibrotic area detected by Masson’s trichrome staining. (D): Quantitative RT-PCR analyses for FN expression in the kidney. (E): Quantitative RT-PCR analyses for COL-I expression in the kidney. (F): Representative western blot images for FN, COL-I and α-SMA. (G): Bar chart showing the FN protein expression normalized by β-actin. (H): Bar chart showing the COL-I protein expression normalized by β-actin. (I): Bar chart showing the α-SMA protein expression normalized by β-actin. *n* = 3–7/group. *p < 0.05. NS: non-significant.

Compared with the sham group, uIRI caused a significant decrease in kidney weight in all uIRI groups, and mice with different ischemic body temperatures developed different degrees of kidney fibrosis. Comparisons between two groups with adjacent temperatures showed that the decrease in kidney weight had no significant difference when the core body temperature increased by 0.5 °C during ischemia (*p* > 0.05), suggesting that the difference of 0.5 °C in core body temperature during ischemia had no significant effect on the change of kidney weight. When core body temperature during the ischemic period had a 1 °C difference, the kidney weight changed significantly (*p* < 0.05), and it gradually decreased as the ischemic body temperature increased (Figure 7(A)).

##### Masson’s trichrome stain

At 8 weeks after uIRI, Masson’s trichrome staining results showed that compared with the sham group, the fibrotic area of kidney tissues in all uIRI groups was significantly increased (*p* < 0.05), indicating that fibrotic outcome occurred in all uIRI groups. Comparisons between two groups with adjacent temperatures showed that the area of kidney fibrosis increased but with no statistical significance when the core body temperature increased by 0.5 °C during ischemia (*p* > 0.05), suggesting that the difference in 0.5 °C in core body temperature during ischemia had no significant effect on kidney fibrosis. For every 1 °C increase in core body temperature during ischemia, the kidney interstitial fibrosis area significantly increased (*p* < 0.05), indicating that the 1 °C increase in core body temperature during ischemia had a significant impact on the kidney interstitial fibrosis, and kidney fibrosis gradually worsened with the increase in core body temperature during ischemia (Figure 7(B,C)).

##### ECM in kidney tissue

The data showed that compared with the sham group, the expressions of FN and COL-I in kidney tissues of all uIRI groups were significantly increased (*p* < 0.05), which was consistent with the results of Masson’s staining. Comparisons between two groups with adjacent temperatures showed that FN and COL-I expressions in the kidney tissues increased by 0.5 °C increase in core body temperature during ischemia, but there was no statistically significant difference (*p* > 0.05), indicating that a 0.5 °C difference in core body temperature during ischemia did not have a significant effect on ECM deposition, while the expression of FN and COL-I increased significantly with a 1 °C increase in core body temperature during ischemia (*p* < 0.05), and they had a more pronounced increase with increasing body temperature. In conclusion, the ECM deposition or the fibrotic outcome in the kidney tissues increased with the higher core body temperature during ischemia (Figure 7(D–H)).

##### Myofibroblasts in kidney tissue

Compared with the sham group, the expressions of α-SMA in kidney tissue of all uIRI groups were significantly increased (*p* < 0.05), suggesting that the myofibroblasts were significantly increased in all uIRI groups. Comparisons between two groups with adjacent temperatures showed that the expression of α-SMA in the kidney increased when core body temperature increased by 0.5 °C during ischemia, but there was no significant difference (*p* > 0.05), suggesting that the difference of 0.5 °C in ischemic body temperature had no significant effect on the changes of kidney myofibroblasts. Moreover, the expression of α-SMA in the kidney tissues significantly increased with a 1 °C increase in ischemic body temperature, and the α-SMA expression increase was more significant with increasing body temperature (*p* < 0.05), suggesting that myofibroblasts changed significantly when the difference in core body temperature during ischemia was 1 °C or more. Along with the increase in core body temperature during ischemia, myofibroblasts in kidney tissues were gradually increased (Figure 7(F,I)).

### Protocol 3: effects of ischemic durations on the AKI-CKD transition

#### Survival rate decreased as the ischemic duration increased

**Figure 8.**
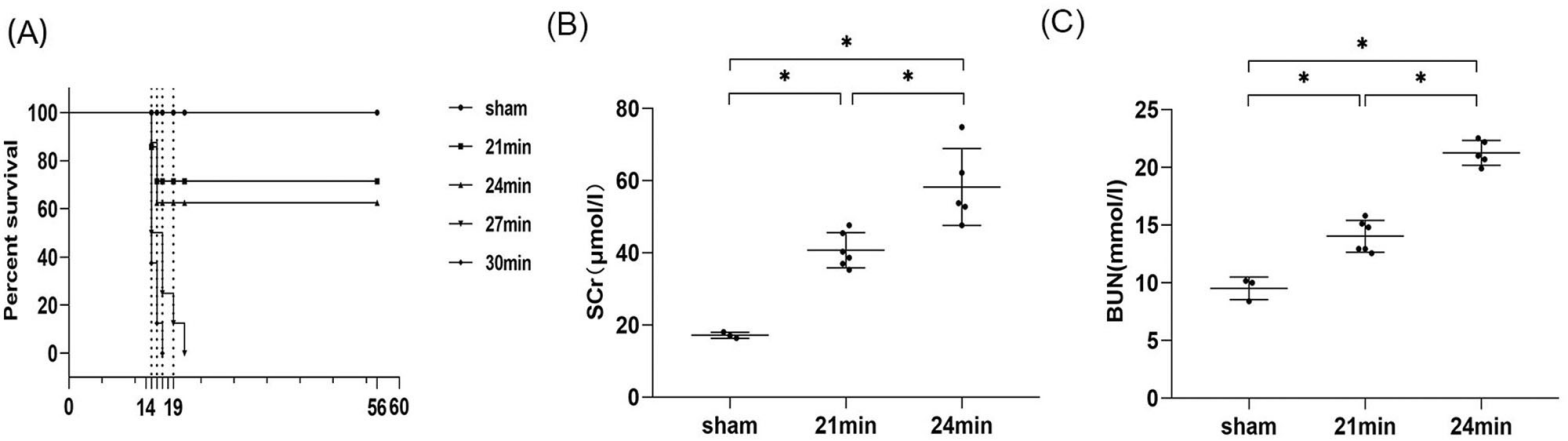
Effects of ischemic durations on survival rate and kidney function in mice with uIRI and delayed contralateral nephrectomy. The left kidney was subjected to IRI on day 0, and right kidney resection was conducted at different ischemic durations (21 min, 24 min, 27 min, and 30 min) with the core body temperature fixed at 37 °C during kidney ischemia, and the right kidney was removed 14 days after the left kidney IRI. (A): The survival of the mice in each group was scored and presented as a percentage. (B and C): Kidney function was estimated by serum creatinine (SCr), and blood urea nitrogen (BUN). *n* = 5–6/group. *p < 0.05.

As mentioned in experiment 3, in order to investigate the effect of ischemic durations on the AKI-CKD transition, the ischemic body temperature was fixed at 37 °C during uIRI, and the contralateral kidney was removed 14 days after uIRI. The results showed that in the 21 min group (the ischemic duration was 21 min), one mouse died 1d and 2d after removal of the contralateral kidney respectively, with a survival rate of 75%, and one more mouse died 2 days after contralateral nephrectomy in the 24 min group, with a survival rate of 62.5%. In the 27 min group, four mice, two mice, one mouse, and one mouse died on 1 day, 3 days, 5 days, and 7 days, respectively, after contralateral nephrectomy, and the survival rate was 0%. Similarly, five mice, two mice, and one mouse died on 1 day, 2 days, and 3 days after contralateral nephrectomy in the 30 min group, and the survival rate was 0%. This indicated that the survival rate decreased as the ischemic duration increased, as all mice in the 27 min group and the 30 min group died 7 days and 3 days, respectively, after contralateral nephrectomy (Figure 8(A)).

#### Kidney function decreased with longer ischemia duration

Data showed that compared with the sham group, both the 21 min group and 24 min groups showed a significant increase in SCr and BUN (*p* < 0.05), indicating kidney function declined. Compared with the 21 min group, the 24 min group showed a more pronounced increase in SCr and BUN (*p* < 0.05), which indicated that the kidney function changed significantly with a 3 min difference in ischemic duration, and a more obvious decrease in kidney function could be seen with the longer ischemic duration (Figure 8(B,C)).

#### Kidney histopathological damage worsened with longer ischemia duration

**Figure 9.**
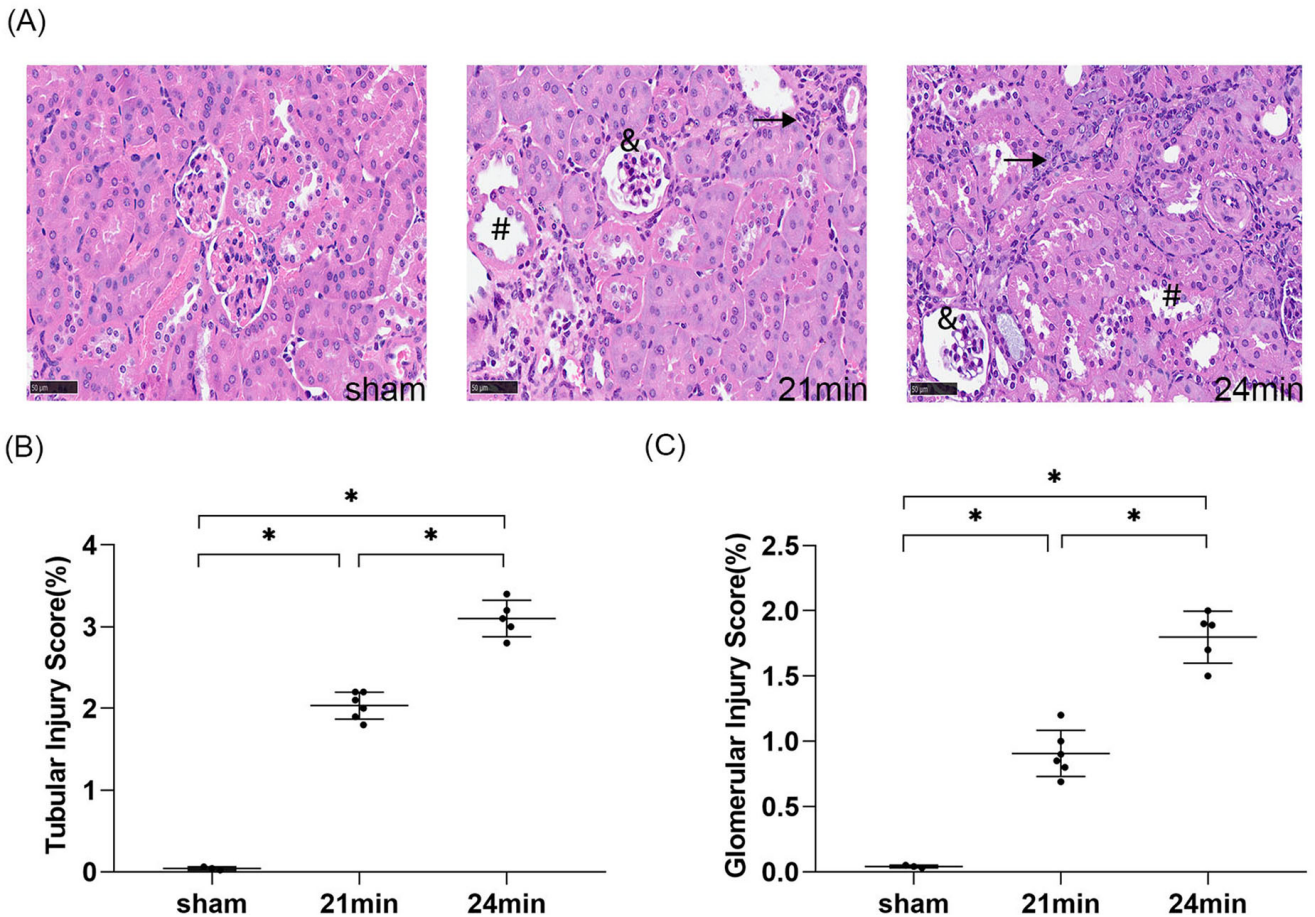
Effects of ischemic durations on kidney histological injury in mice with uIRI and delayed contralateral nephrectomy. The left kidney was subjected to IRI on day 0, and right kidney resection was conducted at different ischemic durations (21 min, 24 min, 27 min, and 30 min) with the core body temperature fixed at 37 °C during kidney ischemia, and the right kidney was removed 14 days after the left kidney IRI. Kidney histological injury was determined by hematoxylin-eosin (HE) staining. (A): Representative HE staining of the injured kidney, which showed the typical histopathological features of post-AKI pathological changes, including different degrees of tubular epithelial cell vacuolar degeneration, brush border detachment, focal disintegration necrosis, dilatation of some tubular lumen (#), kidney tubular epithelial cell edema with variable morphology, and inflammatory cell infiltration (↗). Some glomeruli were wrinkled and sclerotic, with mesangial cell hyperplasia (&) and dilated Bowman’s capsule (magnification, 400×). (B): Semi-quantitation of tubulointerstitial injury. (C): Semi-quantitation of glomerular injury. *n* = 5–6/group. *p < 0.05.

According to HE staining, the kidney tissue structure of the mice in the sham group was normal. Compared with the sham group, the 21 min group and 24 min group had pathological changes in varying degrees, including tubular epithelial cell vacuolar degeneration, brush border detachment, focal disintegration necrosis, dilatation of some tubular lumen, kidney tubular epithelial cell edema with variable morphology, wrinkled and sclerotic glomeruli, mesangial cell hyperplasia and dilated Bowman’s capsule, and inflammatory cell infiltration in the interstitial (Figure 9(A)). Pathological injury scores of kidney tubules and glomeruli were significantly higher in both the 21 min and 24 min groups compared with the sham group (*p* < 0.05), indicating that significant kidney pathological injury developed in both uIRI groups. Furthermore, the 24 min group had higher kidney pathological injury scores and more severe pathological injury compared with the 21 min group (*p* < 0.05) (Figure 9(B,C)), suggesting that kidney pathological kidney injury had significant changes with a 3 min difference in ischemic duration. The longer the duration of ischemia, the more severe the pathological damage of the kidney.

#### Kidney fibrosis worsened with longer ischemia duration

##### Kidney weight

**Figure 10.**
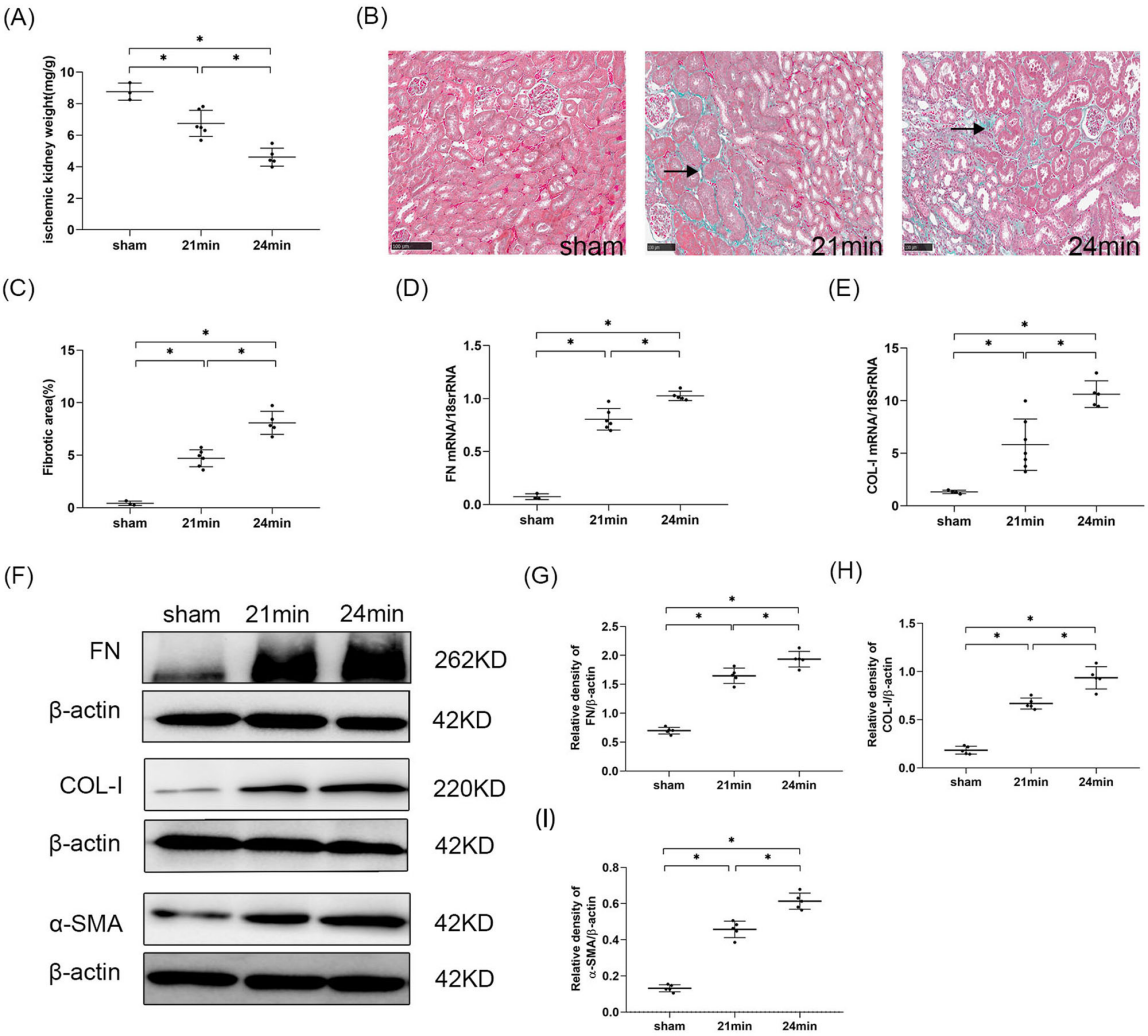
Effects of ischemic durations on kidney fibrosis in mice with uIRI and delayed contralateral nephrectomy. The left kidney was subjected to IRI on day 0, and right kidney resection was conducted at different ischemic durations (21 min, 24 min, 27 min, and 30 min) with the core body temperature fixed at 37 °C during kidney ischemia, and the right kidney was removed 14 days after the left kidney IRI. Kidney fibrosis was assessed by kidney weight, Masson’s trichrome staining, and fibronectin (FN), collagen I (COL-I) and α-smooth muscle actin (α-SMA) expressions. (A): Kidney weight to body weight. (B): Representative Masson’s trichrome staining of the injured kidney, which showed the typical of fibrotic changes (↗) (magnification, 200×). (C): Semi-quantitation of fibrotic area detected by Masson’s trichrome staining. (D): Quantitative RT-PCR analyses for FN expression in the kidney. (E): Quantitative RT-PCR analyses for COL-I expression in the kidney. (F): Representative western blot images for FN, COL-I and α-SMA. (G): Bar chart showing the FN protein expression normalized by β-actin. (H): Bar chart showing the COL-I protein expression normalized by β-actin. (I) Bar chart showing the α-SMA protein expression normalized by β-actin. *n* = 5–6/group. *p < 0.05.

Compared with the sham group, both the 21 min and 24 min groups showed a significant reduction in kidney weight (*p* < 0.05), and the 24 min group had a more prominent kidney weight decrease compared with the 21 min group (*p* < 0.05). These indicated that a 3 min difference in ischemic duration resulted in a significant change in kidney weight, and a more obvious reduction in kidney weight can be seen with longer ischemic duration (Figure 10(A)).

##### Masson’s trichrome stain

Masson’s trichrome staining and semi-quantification of fibrotic area analysis showed that there was no significant interstitial fibrotic change in the sham group, while different degrees of fibrotic changes were seen in the kidney interstitial in both the 21 min and 24 min groups (*p* < 0.05). Compared with the 21 min group, the 24 min group had a higher fibrotic degree of kidney tissue (*p* < 0.05), indicating that kidney fibrosis significantly changed with a 3 min difference in ischemic duration and that more severe kidney fibrosis would become with longer ischemic duration (Figure 10(B,C)).

##### ECM in kidney tissue

Western blot and RT-PCR analysis showed that compared with the sham group, the expressions of FN and COL-I significantly increased in both the 21 min and 24 min groups (*p* < 0.05), indicating that IRI-induced ECM increased significantly in uIRI groups, and the kidney underwent significant fibrotic progression. Compared to the 21 min group, the expressions of FN and COL-I were more pronounced in the kidney tissue in the 24 min group (*p* < 0.05), indicating that the ECM deposition in the kidney tissues changed significantly with a 3 min difference in the ischemic duration, and ECM deposition increased with the prolonged ischemic duration (Figure 10(D–H)).

##### Myofibroblasts in kidney tissue

Western blot analysis showed that compared with the sham group, the expression of α-SMA of the kidney in the 21 min and 24 min groups significantly increased (*p* < 0.05), indicating that myofibroblasts had a significant increase in the kidney in the uIRI groups, and significant fibrotic progression occurred. The expression of α-SMA significantly increased with the 3 min increase in ischemic duration (*p* < 0.05), and more myofibroblasts were found with prolonged ischemic duration (Figure 10(F,I)).

## Discussion

Animal models of kidney IRI, particularly the uIRI model with delayed contralateral nephrectomy, are commonly used to investigate the AKI-CKD transition, but the modeling conditions vary widely among different research [8,10,12,17]. In our study, we assessed the effects of timing of contralateral nephrectomy after uIRI, core body temperatures during ischemia and kidney ischemic durations on the post-AKI fibrotic outcome. The results showed that a longer interval between contralateral nephrectomy and uIRI, higher ischemic body temperature, or longer ischemic duration, caused more severe kidney injury and fibrosis when the other two variables were fixed.

We first observed the effect of time intervals between contralateral nephrectomy and uIRI on the kidney injury. The time intervals between contralateral nephrectomy and uIRI in previous reports varied widely. In some cases, kidney fibrosis of the ischemic kidney was not obvious at 42 days after uIRI when contralateral nephrectomy was conducted 3 days after uIRI [21], and fibrotic outcome could be seen at 56 days after uIRI when contralateral nephrectomy was conducted 7 days after uIRI [10]. Significant fibrotic changes of the ischemic kidney happened at 28 days after uIRI when contralateral nephrectomy was conducted 8 days after uIRI [12], while some researchers found this result only 11 days after uIRI when contralateral kidney was removed 10 days after uIRI [8]. Moreover, some researchers even observed kidney fibrosis at 98 days after uIRI when contralateral kidney was resected at 14 days after uIRI [15]. However, few researchers have investigated the effect of different time intervals between contralateral nephrectomy and uIRI on renal fibrosis. Based on the results reported in the literature and our pre-experiment, we chose 7 days, 10 days and 14 days after uIRI to remove contralateral kidney respectively, with fixed ischemic duration and core body temperature of ischemia during uIRI. The results showed that the survival rate decreased, and the degree of kidney injury, kidney histopathological damage, and kidney fibrosis worsened with the extension of the interval between the two operations. This suggested that the time interval between contralateral nephrectomy and uIRI significantly affected the outcome of the kidney. As far as our current results are concerned, the 3 days’ difference in the time intervals resulted in a significant difference in the severity of AKI progression to CKD. Kidney fibrosis after AKI became more severe with later contralateral nephrectomy. The possible reason for this is that kidney function after IRI is mainly retained by the compensatory work of the contralateral intact kidney, and the blood supply of the injured kidney decreases over time and leads to fibrosis [11,12]. If we remove the contralateral kidney at this point, the function of the injured kidney would not be compensated. The earlier the contralateral intact kidney is removed, the better the compensation effect for the injury will be. On the contrary, when the contralateral nephrectomy is conducted later, the more severe kidney fibrosis will be. Consistent with the previous study [7], uIRI is more likely to induce fibrosis in the injured kidney if the contralateral kidney is preserved, whereas the removal of the intact kidney is conducive to the long-term repair of the IRI kidney [21].

We also found core body temperature during kidney ischemia had an important impact on kidney injury. There are cold and warm kidney IRI. Conducting kidney IRI at 32 °C or below is called the cold one, which is commonly used in kidney transplantation studies [2]. We used the warm ischemia model to investigate AKI-CKD transition, and our pre-experiment showed no obvious fibrotic changes happened when we controlled core body temperature during ischemia at 33 °C, 34 °C or 35 °C. However, most mice could not tolerate high ischemic temperature and died during surgery when it was higher than 39 °C. As the result, we chose 36-38 °C for the ischemic temperature in our study. We observed the effect of core body temperature at 36 °C, 36.5 °C, 37 °C, 37.5 °C and 38 °C on kidney injury with a fixed ischemic duration of 24 min and removal of the contralateral kidney 14 days after uIRI. The results showed that higher core body temperature during kidney ischemia resulted in a lower survival rate, more pronounced deterioration in kidney function and more severe kidney fibrosis. Similar to previous reports [2], the 1 °C difference in core body temperature led to statistically significant changes in kidney function and the fibrotic outcome mentioned above. We found that a 0.5 °C difference in core body temperature during ischemia did not result in a significant difference in fibrosis severity. Consistent with the need to control core body temperature fluctuations within 0.5 °C during ischemia among the groups, which was highlighted by only a few studies [22]. These results provided strong evidence for the temperature control range for IRI modeling. Our group previously also found that if the core temperature was too low during ischemia, the kidneys did not have significant fibrosis manifestations even if the ischemia duration was long enough (results not shown), which also suggested the importance of strict control of ischemic temperature. However, few papers published to date have mentioned core temperature during kidney ischemia, Skrypnyk et al. [12] fixed mice on a water bath system at 38 °C without mentioning the core body temperature during ischemia. Colombaro et al. [14] and Li et al. [16] did not mention the ischemic temperature in their studies, indicating that the effect of ischemic temperature on modeling has not been adequately emphasized.

Finally, we investigated the effect of the ischemic duration on CKD progression after AKI. The ischemic duration described in earlier studies ranges from 15 min [15] to 45 min [10]. When the ischemic duration was less than 21 min in our pre-experiment, no significant fibrotic changes were found. When the ischemic duration was longer than 30 min, all mice died in 24 h after contralateral nephrectomy. Therefore, in our study, the ischemic duration was set at 21–30 min. Ischemic durations were set at 21 min, 24 min, 27 min and 30 min respectively, with the core body temperature at 37 °C and the time interval between contralateral nephrectomy and uIRI of 14 days. As described in the results, all mice in the 27 min and 30 min groups died within 7 days after contralateral nephrectomy, indicating that the injury was severe and exceeded the survival threshold of mice. Fortunately, the effect of ischemic time on kidney injury has now been noted by other research and our results were consistent with the study by Wei et al. [15]. Kidney ischemic duration above 27 min with strict control of the core body temperature at 37 °C during ischemia resulted in fatal injury, and 21 min and 24 min were appropriate ischemic times. Regarding the ischemia times and their corresponding degrees of kidney injury, our results clearly differ from parts of previous studies. In the study of Guan et al., the longest ischemia time was 45 min [10]. According to Li et al., the ischemia time of 35 min induced moderate to severe injury [16]. However, the ischemia time of 30 min in most research caused moderate to severe injury [2,8,12,17,23]. The reason for the above differences may be due to the lack of strict control of core body temperature during ischemia. As far as our current results are concerned, 3 min’ difference in ischemic duration made a significant difference in the severity of AKI progression to CKD with other ischemic conditions fixed, and the longer ischemic duration resulted in a more pronounced CKD progression.

In summary, our results showed that although the uIRI model with delayed contralateral nephrectomy resulted in kidney function impairment, histopathological changes, and fibrotic outcome in most cases, which meant AKI-CKD transition, the interval between contralateral nephrectomy and uIRI, core body temperature during ischemia, and ischemic duration significantly affected the survival rate, kidney function, and kidney fibrosis, i.e., the severity of CKD. Therefore, controlling experimental conditions strictly is very important for the stability and consistency of the animal model, which is the basis for completing later studies and should be of great importance to researchers. Our study also provided a reference for other researchers to successfully establish AKI-CKD models.

The survival rate in our uIRI model with delayed contralateral nephrectomy was somewhat lower than other studies under the same conditions [15]. It’s probably because the difference in laboratory conditions, operators’ techniques and surgical proficiency, experimental environments with different temperatures, temperature control equipment, and micro-arterial clampers, which might affect the results. So a constant environment temperature, and trained and skilled operators are also important to establish a consistent and reliable ischemic AKI model.

Our study also had limitations. Limited by the huge workload, we only studied the effects of contralateral nephrectomy performed on day 7, day 10 and day 14 after uIRI and did not observe the effect of more subtle time interval differences (e.g., day 8 and day 9 after uIRI) when investigating the effect of the time points of contralateral nephrectomy after uIRI on kidney injury. The ischemic durations were only set at 21 min, 24 min, 27 min and 30 min when studying the effect of different ischemic durations on kidney fibrosis, as described in our pre-experimental results and previous reports. The effect of smaller differences in ischemic time (e.g., 22 min, 23 min) on fibrotic outcome was not observed. Therefore, based on the results currently available, we can only draw the conclusion that 3 days’ difference in time interval or 3 min’ difference in time length has a meaningful impact on kidney injury, while further research is still needed to determine the impact of shorter time intervals. A higher core body temperature during ischemia or longer ischemic duration caused higher mortalities when other conditions were fixed. The number of surviving mice was relatively small when the core temperature was above 37 °C or when the ischemic time was longer than 24 min, which may have an impact on the later data analysis. But the results obtained were still statistically significant due to the small differences within the groups, so no additional mice were added.

In conclusion, in the uIRI model with delayed contralateral nephrectomy, kidney fibrosis after AKI becomes more severe as the interval between contralateral nephrectomy and uIRI is prolonged, the core body temperature rises during ischemia, and the ischemic duration increases. The experimental conditions should be strictly controlled in future studies to set a universal standard for the AKI-CKD animal model.

## Author contributions

X.Q. and J.Z. conceived and designed research; J.Z., R.S., and H.L. performed experiments; J.Z., H.L., and R.S. analyzed data; J.Z., J.P., and X.F. interpreted results of experiments; J.Z. L.L. and X.S. prepared figures; J.Z., R.S. and H.L. drafted manuscript; X.Q., C.W. and L.W. edited and revised manuscript; X.Q., J.Z., R.S., H.L., J.P., X.F., L.L., D.N.,Y.H., X.S., C.W., and L.W. approved final version of manuscript.
